# Eyes of Aniso-Axial Length Individuals Share Generally Similar Corneal Biometrics with Normal Eyes in Cataract Population

**DOI:** 10.1155/2020/4760978

**Published:** 2020-10-30

**Authors:** Min Zhang, Tianhui Chen, Michael Deng, Jiahui Chen, Qinghe Jing, Yongxiang Jiang

**Affiliations:** ^1^Department of Ophthalmology and Vision Science, Eye and ENT Hospital of Fudan University, Shanghai, China; ^2^NHC Key Laboratory of Myopia (Fudan University), Key Laboratory of Myopia, Chinese Academy of Medical Sciences and Key Laboratory of Visual Impairment and Restoration of Shanghai, Shanghai, China

## Abstract

**Aims:**

To determine the characteristics of corneal biometrics in eyes from aniso-axial length cataract patients compared with eyes from non-aniso-axial length individuals.

**Methods:**

This is a retrospective case series. Cataract patients with preoperative binocular measurements were recruited. A binocular axial difference of ≥1 mm was considered to indicate aniso-axial length. The anterior segmental biometrics were measured using Pentacam HR (Oculus, Wetzlar, Germany) and IOLMaster 500 (Carl Zeiss Meditec, Jena, Germany). Comparisons of biometrics were made among 4 eye conditions: the longer eyes from aniso-axial length patients, the shorter eyes from aniso-axial length patients, the longer eyes from non-aniso-axial length patients, and the shorter eyes from non-aniso-axial length patients. The aniso-axial length eyes were also stratified into 8 subgroups with axial length (AL) increments of 1 mm, and the biometrics of the subgroups were compared.

**Results:**

There was smaller anterior corneal astigmatism in the shorter aniso-axial length group than those in the longer aniso-axial length group (1.01 ± 0.70 D vs 1.12 ± 0.76 D, *P*=0.031). The longer aniso-axial length eyes had greater anterior corneal steep curvature (44.13 ± 1.69 D vs 43.87 ± 1.69 D, *P*=0.009) and anterior corneal astigmatism (1.12 ± 0.76 D vs 1.02 ± 0.69 D, *P*=0.023) compared with longer non-aniso-axial length subjects. Other corneal biometrics were similar between the aniso-axial length eyes and the non-aniso-axial length eyes. In the longer aniso-axial length group, the posterior corneal aberrations of eyes in the ≥5 mm subgroups were greater than those in the <5 mm subgroups (0.879 ± 0.183 *μ*m vs 0.768 ± 0.178 *μ*m for total aberrations, *P* < 0.001; 0.228 ± 0.086 *μ*m vs 0.196 ± 0.043 *μ*m for high-order aberrations, *P*=0.036; 0.847 ± 0.173 *μ*m vs 0.741 ± 0.179 *μ*m for low-order aberrations, *P*=0.001).

**Conclusion:**

Eyes of aniso-axial length individuals share generally similar corneal biometrics with normal eyes in cataract population. Anterior corneal astigmatism of the longer eyes from the aniso-axial length cataract patients was higher than that of the longer eyes from the non-aniso-axial length individuals. Total posterior corneal aberrations of the longer aniso-axial length eyes increased when the binocular axial difference was over 5 mm.

## 1. Introduction

Anisometropia is a distinct condition of binocular asymmetry—both the eyes of an individual share an identical genetic background and similar environmental exposure but develop significantly different refractive status [1]. It is one of the leading causes of amblyopia, either alone or combined with strabismus [2, 3]. Anisometropia also follows amblyopia caused by either deprivation or strabismus. However, the chronology and effects of anisometropia on ocular biometrics are unclear [4].

The well-accepted standard for anisometropia is a difference of >1.00 diopter (D) of the spherical lens (DS) and/or 1.00 D of the cylinder lens [5, 6]. The comparison of binocular parameters in the anisometropic eye was studied previously, indicating that axial length (AL) was the most important factor in anisometropia [7]. Whether or not other anterior segmental parameters such as corneal power, lens power, and anterior chamber resulting in or related to anisometropia is under debate [7–9].

On the one hand, previous studies have fully analyzed the general relationship between AL and corneal biometrics, indicating flatter cornea and smaller corneal curvature (*K* values) with AL elongation [10]. On the other hand, available anisometropia studies only focused on binocular parameter comparisons in the anisometropia patients rather than comparisons between normal and anisometropia eyes. As the etiology of anisometropia is still unclear and the binocular development is asymmetric in the anisometropic eyes, we are not sure if the anisometropic eyes and the normal eyes follow the same rule of AL-K value interactions. Here, we were supposed to figure out if the corneal biometrics in anisometropic eyes are different from that in nonanisometropic eyes following the AL elongation. We undertook to define the monocular characteristics of anisometropia patients by comparing the ocular biometrics of the asymmetrically developed eyes with those of the normal eyes in a large cohort of cataract patients.

Lens opacity also contributed to anisometropia diagnosis [8]. In cataract population, early asymmetric cataract development (especially nuclear sclerosis) in both eyes was reported to account for about 40% of anisometropia [11]. However, cataract patients have reduced fixation stability and visual acuity, along with the substantial degree of optometry variability [2], and measurement precision of cataract cases needs to be taken into account when diagnosing anisometropia. Also, lens-related factors contributing to anisometropia make no sense for them as a cataract is replaced by intraocular lens (IOL) after surgical treatment. Thus, the clinical significance of preoperative optometry in cataract patients is reduced.

Considering AL plays the most important role in anisometropia [7, 8, 12, 13], is independent of the cataract surgery itself, can be precisely measured in cataract patients [14], and has unparalleled status in IOL power calculation, we assume that “aniso-axial length” is synonymous with “anisometropia.” In this study, it is defined as an individual's binocular axial difference of ≥1 mm. First, according to the Gullstrand eye model, the AL of the eye globe is 24.38 mm [15], and other modified schematic eyes and investigation results have shown this length to be around 24 mm [16, 17]. Second, eyes with AL ≥26 mm or with refractive errors more serious than −6.00 DS are equally considered high myopia [18]. Third, in children aged 6–7 or 12–13 years with anisometropia of ≥1 D, the mean asymmetry of the interocular AL was 0.40 ± 0.40 or 0.60 ± 0.50 mm, respectively [6]. Therefore, an increase in the AL of 1 mm can cause a change in the refractive error at the corneal surfaces of about −3.00 DS. Because other factors may compensate the anisometropia caused by aniso-axial length, we consider that an axial difference of ≥1 mm can be deemed anisometropia, or even serious anisometropia of about ≥3 DS.

Thus, we aimed to (1) compare the binocular corneal biometrics from cataract patients with an axial difference ≥1 mm; (2) compare the corneal biometrics of eyes from cataract patients with an axial difference ≥1 mm to those of eyes from the cataract patients with symmetric binocular ALs; and (3) determine the characteristics of corneal biometrics in aniso-axial length cataract patients.

## 2. Materials and Methods

This was a retrospective case series conducted at the Eye and ENT Hospital of Fudan University, Shanghai, China. It was approved by the Human Research Ethics Committee of the Eye and ENT Hospital of Fudan University (no. 2020103) and complied with the tenets of the Declaration of Helsinki.

All patients were recruited, and the data were collected between September 29, 2016, and August 15, 2018, following the methods described before [19, 20]. In general, cataract patients with ocular comorbidities or history of ocular surgeries or contract lens within 2 weeks were excluded. A rotating Scheimpflug camera (Pentacam HR; Oculus, Wetzlar, Germany) and partial coherence interferometry (IOLMaster 500; Carl Zeiss Meditec, Jena, Germany) were used for data collection. Only patients in whom both eyes were examined were included. Written informed consent was obtained from each patient.

All patients were divided into the aniso-axial length group (binocular axial difference ≥1 mm) and the non-aniso-axial length group (binocular axial difference <1 mm). The two eyes of each patient were separated into the longer one and the shorter one. Each eye from the aniso-axial length group in the longer set and the shorter set got the AL matched with one or two eyes from the non-aniso-axial length group in the longer set and the shorter set, respectively (see more details in Appendix Methods). Corneal astigmatism was defined as “with the rule” (WTR), “against the rule” (ATR), or “oblique,” according to the axis of the corneal steep meridian, as previously described [19].

All continuous data are shown as means ± standard deviations (SD). The normality of continuous data was tested using the Kolmogorov–Smirnov test. All categorical data were compared using the chi-square test. Variables were compared among the longer aniso-axial length, the shorter aniso-axial length, the longer non-aniso-axial length, and the shorter non-aniso-axial length groups using analyses of variance (ANOVA) and a post hoc analysis with the LSD correction. Studied variables included the following: (1) anterior corneal curvature (flat and steep radius of curvature, *r*1 and *r*2), anterior and posterior corneal curvature values (flat and steep power of curvature, *K*1 and *K*2; average radius and power of curvature defined as the central radius of curvature in the steep direction/central radius of curvature in the flat direction using *n* = 1.3375, *Rm* and *Km*), and astigmatism; (2) the root mean square of the anterior, posterior, and total corneal low-order aberrations (LOAs), high-order aberrations (HOAs), and total aberrations (TAs); (3) the anterior, posterior, and total corneal Zernike polynomial coefficients of the third-order aberrations (vertical coma, horizontal coma, vertical trefoil, and oblique trefoil) and the primary spherical aberrations (SAs); and (4) the central corneal thickness (CCT). Except for *r*1, *r*2 and CCT reported using IOLMaster 500, others were obtained using Pentacam HR.

All aniso-axial length eyes in both the shorter and longer sets were also divided into eight subgroups based on the axial difference in increments of 1 mm (1-2, 2-3, 3-4, 4-5, 5-6, 6-7, 7-8, and 8-9 mm, where a value of 1 mm is included in the 1-2 mm subgroup, and similarly for other values that fall at the boundary between two subgroups). The variables were compared again among all subgroups in the two sets with ANOVA and post hoc LSD correction, respectively. Data analyses were performed using SPSS 26.0 (SPSS, IBM Corp., Armonk, NY, USA). A *P* value < 0.05 was considered to indicate statistical significance.

## 3. Results

**Figure 1:**
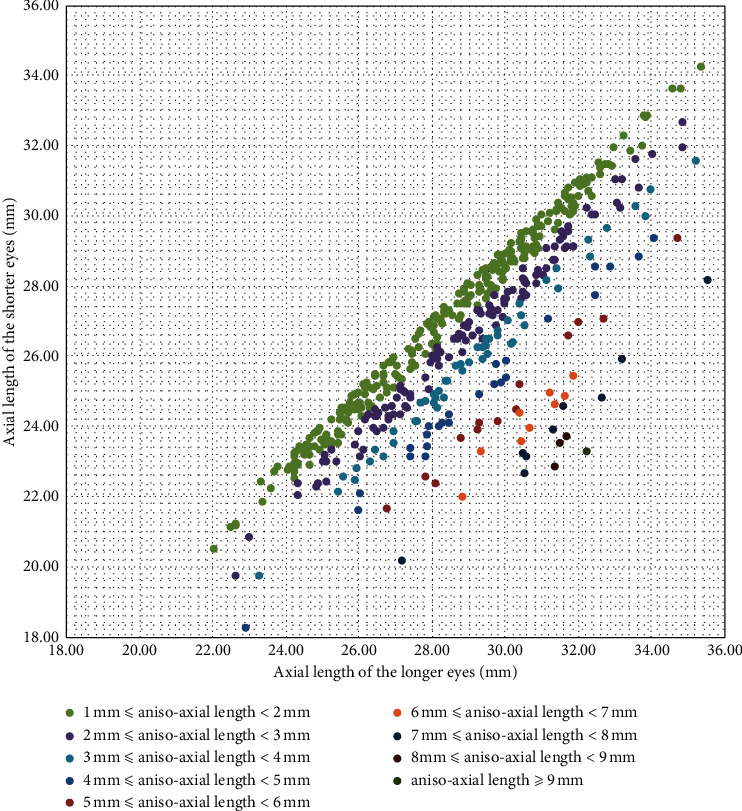
Distributions of the binocular axial lengths of 505 aniso-axial length patients. Every dot in the figure represents one person with a binocular axial difference ≥1 mm. The horizontal axis indicates the axial length of the relative longer eye in each patient, and the vertical axis indicates the axial length of the relative shorter eye.

In total, anterior segmental biometrics of 10,094 eyes from 6747 cataract patients were available and 3347 patients had both eyes examined. Among them, 2842 patients had an absolute difference in the AL of <1 mm between their two eyes. The distribution of the AL of the remaining 505 aniso-axial length patients is shown in Figure 1; among these patients, 16 were excluded because of their young age (<20 years old). Ultimately, 489 aniso-axial length patients were recruited, together with 564 shorter non-aniso-axial length eyes and 597 longer non-aniso-axial length eyes (Table 1). The ≥9 mm subgroup had only one patient and was excluded in statistical analyses among subgroups.

**Figure 2:**
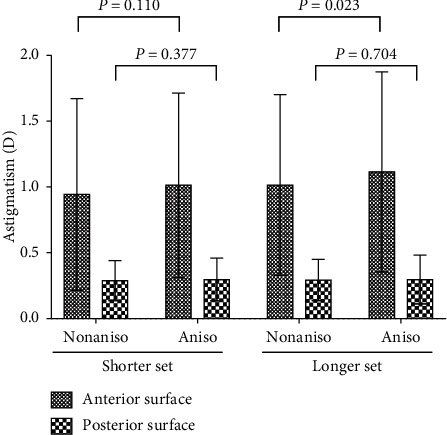
Comparison of astigmatism among the aniso-axial length groups and the non-aniso-axial length groups in the shorter set or the longer set. Non-aniso = non-aniso-axial length group, Aniso =  aniso-axial length group. *P* values were reported by post hoc LSD correction.

Only one statistically significant difference between the shorter and longer eyes in the aniso-axial length group: anterior corneal astigmatism (1.01 ± 0.70 D vs 1.12 ± 0.76 D, *P*=0.031) was detected. Anterior corneal *K*2 and astigmatism were higher in the longer aniso-axial length group compared with the longer non-aniso-axial length group (44.13 ± 1.69 D vs 43.87 ± 1.69 for the anterior corneal *K*2, *P*=0.009; 1.12 ± 0.76 D vs 1.02 ± 0.69 D for the anterior corneal astigmatism, *P*=0.023; Table 2 and Figure 2). The division of the total corneal astigmatism into WTR, ATR, or oblique astigmatism was similar (*P*=0.569; Table 3).

No significant differences were detected between longer or shorter eyes from aniso-axial length or non-aniso-axial length individuals in HOAs, coma, or trefoil. The SAs were slightly higher in the aniso-axial length group and highest in the shorter aniso-axial length group (ANOVA, all *P* > 0.100). The anterior corneal, posterior corneal, and total corneal SAs of the shorter aniso-axial length eyes were 0.34 ± 0.12 *μ*m, −0.13 ± 0.03 *μ*m, and 0.31 ± 0.12 *μ*m, respectively, and those of the longer aniso-axial length eyes were 0.33 ± 0.12 *μ*m, −0.13 ± 0.03 *μ*m, and 0.30 ± 0.12 *μ*m, respectively (Table 4). The anterior and posterior corneal SAs did not differ significantly among the four groups. We found wide distributions of SAs in the aniso-axial length groups and the non-aniso-axial length groups (Table 4). By the way, a total of 13 eyes with negative SAs were detected, 7 eyes from the non-aniso-axial length group and 6 eyes from the aniso-axial length group. Their specific corneal biometrics are presented in Appendix Table A1.

**Figure 3:**
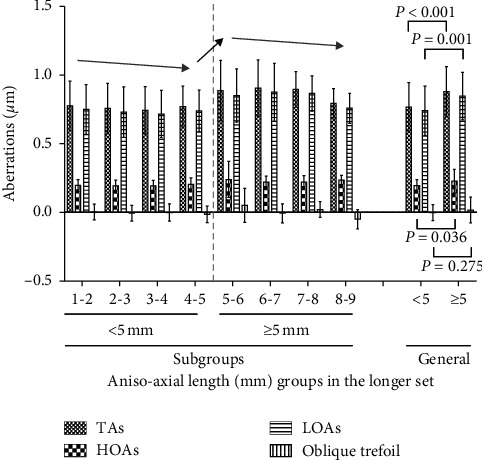
Distribution of posterior corneal aberrations among the eight subgroups in the longer set. TAs = total aberrations; HOAs = high-order aberrations; LOA = low-order aberrations. For the aniso-axial length subgroups, a value of 1 mm is included in the 1-2 mm subgroup and similarly for other values that fall at the boundary between two subgroups. The *P* values for TAs, HOAs, LOAs, and oblique trefoil with analyses of variance among the eight aniso-axial length subgroups in the longer set were 0.025, 0.005, 0.039, and 0.040, respectively. The *P* values for TAs, HOAs, LOAs, and oblique trefoil using independent Student's *t* test between aniso-axial length eyes in the longer set with and without AL ≥5 mm were <0.001, 0.036, 0.001, and 0.275, respectively.

There were 266, 114, 50, 25, 13, 8, 9, and 3 aniso-axial length patients with axial differences of 1-2, 2-3, 3-4, 4-5, 5-6, 6-7, 7-8, and 8-9 mm, respectively. ANOVA of the corneal biometrics detected significant differences in the posterior corneal TAs (*P*=0.025), posterior corneal HOAs (*P*=0.005), posterior corneal LOAs (*P*=0.039), and posterior corneal oblique trefoil (*P*=0.040) among subgroups in the longer set, whereas there were no significant difference among subgroups in the shorter set. The distributions of the variables in the eight subgroups of the longer set are shown in Figure 3. Post hoc analysis with LSD correction indicated an increase of posterior corneal TAs, HOAs, LOAs, and oblique trefoil at an axial difference of 5 mm (*P* < 0.05 of comparisons between the subgroups with an axial difference of <5 mm and ≥5 mm. Exact standard errors, mean differences, or *P* values were not shown) for all four variables. General comparisons in the longer set between eyes with an axial difference < 5 mm and ≥5 mm also indicated statistical significance in TAs (0.879 ± 0.183 *μ*m vs 0.768 ± 0.178 *μ*m, *P* < 0.001), HOAs (0.228 ± 0.086 *μ*m vs 0.196 ± 0.043 *μ*m, *P*=0.036), and LOAs (0.847 ± 0.173 *μ*m vs 0.741 ± 0.179 *μ*m, *P*=0.001 ). However, the same 5 mm cut-off was not found among subgroups in the analysis of ALs with ANOVA (Appendix Table A2).

## 4. Discussion

Anisometropia is the condition in which an individual's eyes have significant binocular refractive differences. Shapira et al. [21] found that the more myopic eye of anisometropic patients before refractive surgery yielded lower predictability and accuracy in terms of surgical outcomes and tended to be overcorrected, whereas the less myopic eye had similar outcomes as the isometropic control eyes. This indicated that there were some unknown characteristics of anisometropic eyes. We tried to address this problem with a cataract population in this study.

Patients in this study had unusually long axial length (26.56 ± 2.77 mm in the shorter aniso-axial length group and 28.92 ± 2.71 mm in the longer aniso-axial length group) and were incredibly young (58.01 ± 10.57 years old in the aniso-axial length group). This is consistent with our previous publication based on the same cataract population, reporting 26.65% patient with an AL >26 mm among 6747 eyes from 6747 patients [20]. There are two reasons for such a large proportion of high myopia in this population. First, urban China has a high prevalence of high myopia. In survey among university students in Shanghai, which is the most advanced area in China and the location of our hospital, the prevalence of high myopia was 19.5% in 2012 [22]. Second, instead of an epidemiological investigation, our study population is a retrospective hospital-based clinical research. Our hospital is one of the top specialized hospitals in ophthalmology in China, high-myopia patients come to our hospital in great numbers. Thus, the percentage of high myopia in our cataract patients rises to 25% in this study. As high-myopia patients tended to develop cataract early [23], it was reasonable to have relatively young cataract population compared with the general population.

In this study, using a definition of aniso-axial length/anisometropia as a binocular axial difference of 1 mm (about 3 D), the percentage of aniso-axial length patients was 15.09% (505/3347). Most aniso-axial length patients had 1-2 D asymmetry in AL between the two eyes (275/505 = 54.46% with no age limit; Figure 1). Deng et al. [24] reported a prevalence of anisometropia of >20% in elderly patients of >1 D spherical refraction. Our comparatively lower fraction seems credible as stricter definition of aniso-axial length/anisometropia was applied.

Among all the aniso-axial length patients, we detected greater tendencies for female patients to develop aniso-axial length and for the right eye to be the longer one, which are consistent with the findings of Linke et al. [25] and Singh et al. [9], respectively. Many studies support the proposition that the dominant eye is the more myopic in patients with myopic anisometropia [26–29]. The dextromanuality might contribute to the greater proportion of myopic right eyes and right dominance of eyes [30]. So, it is reasonable to find the right eyes as the longer ones in aniso-axial length individuals with the dextromanuality of Chinese.

In this study, we found no significant differences in corneal biometrics between the two eyes of aniso-axial length patients, except anterior corneal astigmatism (1.01 ± 0.70 D in the shorter eyes vs 1.12 ± 0.76 D in the longer eyes, *P*=0.031), and only slightly greater aberrations in the shorter eyes compared with the longer eyes of aniso-axial length patients (all *P* > 0.05 for anterior corneal, posterior, and total cornea; data not shown). These findings are roughly consistent with those of previous studies [1, 31] of anisomyopes, which have reported similar levels of aberrations between the two eyes or slightly higher levels in the less myopic eye.

The focus of this study was the comparison of the longer and shorter eyes of aniso-axial length patients with the AL-matched eyes of non-aniso-axial length patients. To our surprise, when examining the anterior corneal surface, only *K*2 differed between the aniso-axial length and non-aniso-axial length groups (44.13 ± 1.69 D vs 43.87 ± 1.69 D, *P*=0.009). *K*1, *K*2, *Rm*, and *Km* of the posterior corneal surface in both the longer set and the shorter set had significant lower values (larger absolute values when negative) in the aniso-axial length group than in the non-aniso-axial length group (Table 2). This might make some compensation for the binocular refractive imbalance in aniso-axial length patients. Though the contribution of the posterior corneal curvature is small [32], its effects on total corneal refractive status should not be neglected casually, especially in aniso-axial length patients.

Because of the difference in the corneal curvature, we expected to find a difference in corneal astigmatism between eyes from the aniso-axial length and the non-aniso-axial length groups. Astigmatism was greater in the longer aniso-axial length group than those in the longer non-aniso-axial length group, but only significant in anterior corneal astigmatism (1.12 ± 0.76 D vs 1.02 ± 0.69 D for the anterior corneal surface, *P*=0.023 ; and 0.30 ± 0.19 D vs 0.29 ± 0.16 D for the posterior corneal surface, *P*=0.377; Figure 2). Though no difference in posterior corneal astigmatism may hold true in the means, it cannot be so in individuals, as posterior corneal curvatures (*K*1, *K*2, and *Km*) were smaller in the aniso-axial length group than those in the non-aniso-axial length group. Again, the contribution of posterior corneal surface to the total corneal refractive status should be paid attention to in aniso-axial length individuals.

Though statistically significant, the difference of anterior corneal astigmatism between the longer aniso-axial length and the non-aniso-axial length was only 0.10 D. This is partially consistent with previous studies that have shown that the asymmetry of the ocular refractive status does not seriously affect the cornea, while some have reported statistically significant effects [1, 8, 33, 34]. It also confirmed a smaller contribution of corneal astigmatism to the anisometropia compared with defocus (where AL plays an important role), supporting the rationale of defining aniso-axial length/anisometropia as AL asymmetry. As we matched these eyes from the aniso-axial length patients solely by AL to the eyes of other non-aniso-axial length patients, the mean corneal powers were also very similar and the difference in mean corneal astigmatism was small. And most of the other parameters are similar, which seems to indicate, a little indirectly, that axial length is the main thing that varies.

Of the SAs and other HOAs, the total corneal SA of the aniso-axial length eyes remained stable at 0.302 ± 0.120 *μ*m in the longer set and 0.306 ± 0.125 *μ*m in the shorter set and did not differ from those of the non-aniso-axial length patients (both *P* > 0.050). The tendency of SA to be lower in the shorter set than in the longer aniso-axial length group is consistent with our previous study [19] of 502 eyes with cataract and axially high myopia, and 1500 eyes with age-related cataracts (0.281 ± 0.207 *μ*m vs 0.314 ± 0.153 *μ*m for total corneal SA, respectively, *P* < 0.001). The implantation of an aspheric IOL with −0.20 *μ*m to achieve an outcome of +0.10 *μ*m SA would be the preferred option, as described before [19]. The range of total corneal SAs in the non-aniso-axial length group was −0.154 to 0.702 *μ*m (one abnormal case excluded), and the distribution of SAs of the aniso-axial length group (from −0.365 to 0.742 *μ*m) was wide. Therefore, customized SA correction may be the best option for the aniso-axial length cataract population. Although a negative ocular SA might lead to axial elongation, it seems that the incidence of negative corneal SA does not correlate with the monocular AL, regardless of whether there is a binocular axial difference (Appendix Table A1).

ANOVA with LSD post hoc correction of the corneal biometrics in the subgroups with various axial differences in the longer set indicated a clear cut-off at 5 mm for the posterior corneal TAs, HOAs, LOAs, and oblique trefoil (Figure 3). This is inconsistent with an axial cut-off at 5 or 7 mm in the longer set (Appendix Table A2). Our previous studies [19, 35] suggestthat the corneal aberrations of highly myopic cataract patients and those with age-related cataract differed only slightly and these are insufficient to explain these results. More care must be taken in future clinical studies, and more attention should be paid to posterior corneal aberrations.

The first limitation of this study was its retrospective design without the inclusion of IOL formulae or postoperative refractive outcomes. However, this study is a foundational analysis for a later study, detecting potential differences in refraction-affecting anterior segmental biometrics. The second limitation was that no case with binocular astigmatism difference (especially total corneal astigmatism) was studied because we focused on the asymmetry of AL. Further analyses based on binocular asymmetry of the total corneal astigmatism and inconsistencies its axis/division are required to provide general indications for toric IOL implantation in aniso-astigmatism patients.

In conclusion, eyes of aniso-axial length individuals share generally similar corneal biometrics with normal eyes in cataract population. Anterior corneal astigmatism of the longer eyes from the aniso-axial length cataract patients was higher than that of the longer eyes from the non-aniso-axial length individuals. Total posterior corneal aberrations of the longer aniso-axial length eyes increased when the binocular axial difference was over 5 mm.

## Figures and Tables

**Table 1:** Demographic data of patients included in the study.

	Shorter set			Longer set			Total
	Non-aniso-axial length (*N* = 564)	Aniso-axial length (*N* = 489)	*P* value	Non-aniso-axial length (*N* = 597)	Aniso-axial length (*N* = 489)	*P* value	
Gender based on eyes (male/female)†	268/296	167/322	<0.001^*∗*^	261/336	167/322	0.001^*∗*^	863/1276
Age (years)	57.49 ± 10.28	58.01 ± 10.57	0.415	57.88 ± 10.41	58.01 ± 10.57	0.836	57.84 ± 10.44
Axial length (mm)	26.72 ± 2.71	26.56 ± 2.77	>0.999	28.68 ± 2.85	28.92 ± 2.71	0.890	27.73 ± 2.96

^*∗*^ *P* < 0.05. †Chi-square test.

**Table 2:** Comparisons of corneal biometrics between non-aniso-axial length and aniso-axial length patients in both the shorter and longer sets.

	Shorter set			Longer set			*P* value††
	Non-aniso-axial length	Aniso-axial length	*P* value†	Non-aniso-axial length	Aniso-axial length	*P* value†	
CCT (mm)	544.07 ± 31.88	539.48 ± 32.05	0.018^*∗*^	543.51 ± 30.43	539.92 ± 31.29	0.061	0.028^*∗*^
*K*2 F (D)	43.80 ± 1.63	44.02 ± 1.63	0.033^*∗*^	43.87 ± 1.69	44.13 ± 1.69	0.009^*∗*^	0.005^*∗*^
*r*2 F (mm)	7.66 ± 0.29	7.62 ± 0.29	0.010^*∗*^	7.64 ± 0.30	7.60 ± 0.28	0.007^*∗*^	0.001^*∗*^
*K*1 B (*D*)	−6.16 ± 0.26	−6.19 ± 0.26	0.025^*∗*^	−6.16 ± 0.26	−6.19 ± 0.27	0.047^*∗*^	0.029^*∗*^
*K*2 B (*D*)	−6.44 ± 0.29	−6.49 ± 0.27	0.010^*∗*^	−6.46 ± 0.29	−6.49 ± 0.28	0.054	0.013^*∗*^
*RmB* (mm)	6.36 ± 0.26	6.32 ± 0.25	0.015^*∗*^	6.36 ± 0.27	6.32 ± 0.26	0.042^*∗*^	0.017^*∗*^
*KmB* (mm)	−6.30 ± 0.26	−6.34 ± 0.25	0.012^*∗*^	−6.30 ± 0.27	−6.33 ± 0.26	0.051	0.017^*∗*^
Astigmatism *F* (D)	0.94 ± 0.73	1.01 ± 0.70	0.110	1.02 ± 0.69	1.12 ± 0.76	0.023^*∗*^	0.002^*∗*^
TA *F* (*μ*m)	2.30 ± 0.75	2.38 ± 0.68	0.103	2.33 ± 0.90	2.47 ± 0.78	0.002^*∗*^	0.002^*∗*^
LOA *F* (*μ*m)	2.23 ± 0.74	2.30 ± 0.67	0.106	2.25 ± 0.88	2.40 ± 0.77	0.002^*∗*^	0.002^*∗*^
TA cornea (*μ*m)	2.03 ± 0.72	2.10 ± 0.67	0.148	2.03 ± 0.88	2.18 ± 0.76	0.002^*∗*^	0.005^*∗*^
LOA cornea (*μ*m)	1.95 ± 0.72	2.02 ± 0.65	0.151	1.95 ± 0.86	2.10 ± 0.75	0.002^*∗*^	0.004^*∗*^

†Post hoc LSD correction; ††analyses of variance. ^*∗*^*P* < 0.05. *F* = anterior corneal surface; *B* = posterior corneal surface; Corneal = total cornea; *K*1 = flat power of curvature in the center of anterior surface using *n* = 1.3375 on a ring in 15° around the corneal apex; *K*2 = steep power of curvature in the center of anterior surface using *n* = 1.3375 on a ring in 15° around the corneal apex; *Rm* = average radius of curvature (central radius of curvature in the steep direction/central radius of curvature in the flat direction); *Km* = average power of curvature using *n* = 1.3375; LOAs = low order aberrations; TAs = total aberrations. Only *r*2 and CCT were reported using IOLMaster 500, and others were obtained using Pentacam HR.

**Table 3:** Distributions of total corneal astigmatism divisions in the non-aniso-axial length group and the aniso-axial length group of the two sets.

	Shorter set		Longer set		Total
	Non-aniso-axial length	Aniso-axial length	Non-aniso-axial length	Aniso-axial length	
WTR	297 (52.66%)	250 (51.12%)	308 (51.59%)	238 (48.67%)	1093
ATR	172 (30.50%)	156 (31.90%)	183 (30.65%)	170 (34.76%)	681
Oblique	95 (16.84%)	83 (16.97%)	106 (17.76%)	81 (16.56%)	365
Total	564 (100%)	489 (100%)	597 (100%)	489 (100%)	2139

WTR = with-the-rule astigmatism; ATR = against-the-rule astigmatism; oblique = oblique astigmatism. Percentages of astigmatism division in each group were shown in brackets. *P* > 0.05 using the chi-square test.

**Table 4:** Distributions of total, anterior, and posterior corneal SAs in different groups.

Groups	SAs	Minimum (*μ*m)	Maximum (*μ*m)	Mean ± SD (*μ*m)
Aniso, longer (*N* = 489)	Total cornea	−0.118	0.742	0.303 ± 0.120
	Posterior corneal surface	−0.235	−0.016	−0.128 ± 0.034
	Anterior corneal surface	−0.102	0.768	0.334 ± 0.116

Non, longer (*N* = 597)	Total cornea	−0.133	0.687*∗*	0.295 ± 0.151
	Posterior corneal surface	−0.229	−0.021	−0.132 ± 0.034
	Anterior corneal surface	−0.180	0.751*∗*	0.331 ± 0.148

Aniso, shorter (*N* = 489)	Total cornea	−0.365	0.681	0.306 ± 0.125
	Posterior corneal surface	−0.224	−0.011	−0.128 ± 0.035
	Anterior corneal surface	−0.286	0.695	0.337 ± 0.118

Non, shorter (*N* = 564)	Total cornea	−0.154	0.702	0.293 ± 0.117
	Posterior corneal surface	−0.223	−0.018	−0.131 ± 0.035
	Anterior corneal surface	−0.099	0.698	0.328 ± 0.112

SAs = spherical aberrations; Aniso = aniso-axial length group; Non = non-aniso-axial length group; shorter = shorter set; longer = longer set; SD = standard deviation. The *P* values of total posterior and anterior corneal SAs with analyses of variance were >0.05 among the four groups. *∗*One non-aniso-axial length eye in the longer set with anterior corneal SA = 2.767 *μ*m, posterior corneal SA = −0.097* μ*m, and total corneal SA = 2.748 *μ*m was considered abnormal and was not listed as the maximum.

## Data Availability

Raw data were generated at the Eye and ENT Hospital of Fudan University, Shanghai, China. Derived data supporting the findings of this study are available from the corresponding author on request.

## Additional Points

*Synopsis*. Eyes of aniso-axial length individuals share generally similar corneal biometrics with normal eyes in cataract population. Anterior corneal astigmatism of the longer eyes from the aniso-axial length cataract patients was higher than that of the longer eyes from the non-aniso-axial length individuals.

## Supplementary Materials

Supplementary Materials Appendix methods: axial length match. Appendix Table A1: patients with negative total corneal SAs in the advanced analysis. Appendix Table A2: comparison of axial lengths among the different binocular axial difference subgroups of the longer aniso-axial length set.
