# Supplementary material for: Eyes of Aniso-Axial Length Individuals Share Generally Similar Corneal Biometrics with Normal Eyes in Cataract Population
Source: J Ophthalmol. 2020 Oct 30;2020:4760978. doi: 10.1155/2020/4760978 (PMC7648247; doi:10.1155/2020/4760978)
Supplement: Supplementary Materials — Appendix methods: axial length match. Appendix Table A1: patients with negative total corneal SAs in the advanced analysis. Appendix Table A2: comparison of axial lengths among the different binocular axial difference subgroups of the longer aniso-axial length set. [file 4760978.f1.docx]

**Appendix Methods: Axial length match.**

In each set (the longer set or the shorter set), all the eyes were arranged according to their axial lengths, from longest to shortest. The eyes from aniso-axial length patients were all retained and highlighted. The eyes of the nonaniso-axial length subjects were included in the analysis only when located just upside or downside of the eyes of aniso-axial length patients. Sometimes only one eye from the nonaniso-axial length stuck between the eyes from the aniso-axial length. Therefore, not every eye from the aniso-axial length got two (one upside and one downside) eyes from the nonaniso-axial length match, some shared the eyes from the nonaniso-axial length (for example, two eyes from the aniso-axial length with quite close axial length shared the only one eye from the nonaniso-axial length stuck between these two eyes from the aniso-axial length). The purpose of the axial length match was to develop a new nonanisometropic group sharing statistically insignificant difference with the aniso-axial length group in axial length and demographic. This new nonaniso-axial length group is used for the statistical analyses.

We have to highlight that all eyes from the aniso-axial length were included in final analysis. However, at the time point of longer and shorter set separation, eyes from the nonaniso-axial length subjects were labeled with the “longer” or “shorter” instead of “the longer eye in Patient A” or “the shorter eye compared with the other eye in Patient A”. Two eyes from one nonaniso-axial length subject were not necessarily included or excluded by the match at the same time as they were not linked to each other anymore. Thus, the axial length-match eyes from the nonaniso-axial length were still included who got only one eyes available for analysis.

**Appendix Table A1. Patients with negative total corneal SAs in the advanced analysis.**

| No. | Gender | Age | Eye | Axial length (mm) | K1 F (D) | K2 F (D) | K1 B (D) | K2 B (D) | Astig F (D) | Astig B (D) | SA F (μm) | SA B (μm) | SA Cornea (μm) | Group |
| --- | --- | --- | --- | --- | --- | --- | --- | --- | --- | --- | --- | --- | --- | --- |
| 1 | Male | 29 | Left | 22.72 | 42.4 | 49.0 | -6.0 | -7.1 | 6.6 | 1.1 | 0.083 | -0.208 | -0.011 | Non, Shorter |
| 2 | Male | 67 | Right | 24.26 | 42.8 | 44.1 | -6.2 | -6.6 | 1.2 | 0.4 | -0.040 | -0.161 | -0.095 | Non, Shorter |
| 3 | Male | 47 | Left | 24.99 | 40.9 | 45.3 | -6.0 | -6.8 | 4.4 | 0.8 | -0.180 | -0.042 | -0.133 | Non, Longer |
| 4 | Female | 59 | Left | 28.29 | 44.0 | 44.4 | -5.9 | -6.1 | 0.4 | 0.2 | -0.122 | -0.094 | -0.129 | Aniso, Shorter |
| 5 | Female | 45 | Right | 28.78 | 42.6 | 42.8 | -6.0 | -6.1 | 0.2 | 0.1 | 0.016 | -0.128 | -0.024 | Non, Shorter |
| 6 | Female | 77 | Right | 29.77 | 43.8 | 44.0 | -6.1 | -6.3 | 0.2 | 0.2 | -0.102 | -0.109 | -0.118 | Aniso, Longer |
| 7 | Female | 53 | Left | 30.11 | 42.1 | 43.7 | -6.1 | -6.2 | 1.6 | 0.2 | -0.032 | -0.107 | -0.053 | Non, Longer |
| 8 | Male | 57 | Right | 30.14 | 44.8 | 45.8 | -6.2 | -6.5 | 1.0 | 0.3 | -0.019 | -0.143 | -0.057 | Aniso, Shorter |
| 9 | Female | 59 | Right | 30.91 | 44.0 | 44.0 | -6.0 | -6.2 | 0.0 | 0.2 | -0.052 | -0.096 | -0.062 | Aniso, Longer |
| 10 | Male | 45 | Right | 31.91 | 42.4 | 45.8 | -5.7 | -6.2 | 3.4 | 0.5 | -0.286 | -0.170 | -0.365 | Aniso, Shorter |
| 11 | Female | 42 | Right | 31.98 | 39.2 | 41.1 | -5.7 | -6.0 | 1.9 | 0.2 | 0.014 | -0.093 | -0.011 | Non, Longer |
| 12 | Female | 40 | Left | 32.58 | 41.9 | 44.3 | -5.9 | -6.5 | 2.5 | 0.6 | -0.099 | -0.144 | -0.154 | Non, Shorter |
| 13 | Male | 45 | Left | 33.00 | 42.3 | 44.9 | -5.8 | -6.3 | 2.7 | 0.5 | -0.017 | -0.165 | -0.092 | Aniso, Longer |

Eyes no. 4 and no. 9 were the two eyes of one patient. Eyes no. 10 and no. 13 were the two eyes of one patient. Other eyes are from different individuals.

Aniso = the aniso-axial length groups; non = the nonaniso-axial length groups; shorter = the shorter set; longer = the longer set; F = anterior corneal surface; B = posterior corneal surface; cornea = total cornea; K1 = flat radius of curvature in the center of anterior surface using n = 1.3375 on a ring in 15° around the corneal apex; K2 = steep radius of curvature in the center of anterior surface using n = 1.3375 on a ring in 15° around the corneal apex; astig = astigmatism

**Appendix Table A2. Comparison of axial lengths among the different binocular aixal difference subgroups of the longer aniso-axial length set.**

| Subgroup 1 | Subgroup 2 | SE | Mean Difference | *P* value^†^ | 95% Confidence Interval | |
| --- | --- | --- | --- | --- | --- | --- |
|  |  |  |  |  | **Lower Bound** | **Upper Bound** |
| 1-2 mm | **2-3 mm** | -0.544 | 0.309 | 0.079 | -1.150 | 0.063 |
| 1-2 mm | **3-4 mm** | -0.755 | 0.426 | 0.076 | -1.590 | 0.080 |
| 1-2 mm | **4-5 mm** | -0.732 | 0.578 | 0.205 | -1.866 | 0.401 |
| 1-2 mm | **5-6 mm** | -1.658 | 0.784 | 0.035^*^ | -3.197 | -0.120 |
| 1-2 mm | **6-7 mm** | -2.400 | 0.991 | 0.016^*^ | -4.343 | -0.456 |
| 1-2 mm | **7-8 mm** | -2.991 | 0.936 | 0.001^*^ | -4.827 | -1.155 |
| 1-2 mm | **8-9 mm** | -3.054 | 1.603 | 0.057 | -6.199 | 0.091 |
| 2-3 mm | **3-4 mm** | -0.211 | 0.468 | 0.652 | -1.130 | 0.708 |
| 2-3 mm | **4-5 mm** | -0.188 | 0.610 | 0.757 | -1.385 | 1.008 |
| 2-3 mm | **5-6 mm** | -1.115 | 0.808 | 0.168 | -2.700 | 0.471 |
| 2-3 mm | **6-7 mm** | -1.856 | 1.010 | 0.066 | -3.837 | 0.126 |
| 2-3 mm | **7-8 mm** | -2.447 | 0.956 | 0.011 | -4.323 | -0.571 |
| 2-3 mm | **8-9 mm** | -2.510 | 1.615 | 0.120 | -5.679 | 0.658 |
| 3-4 mm | **4-5 mm** | 0.023 | 0.676 | 0.973 | -1.304 | 1.350 |
| 3-4 mm | **5-6 mm** | -0.903 | 0.860 | 0.293 | -2.590 | 0.783 |
| 3-4 mm | **6-7 mm** | -1.645 | 1.051 | 0.118 | -3.708 | 0.418 |
| 3-4 mm | **7-8 mm** | -2.236 | 1.000 | 0.026^*^ | -4.198 | -0.274 |
| 3-4 mm | **8-9 mm** | -2.299 | 1.641 | 0.161 | -5.520 | 0.921 |
| 4-5 mm | **5-6 mm** | -0.926 | 0.944 | 0.327 | -2.778 | 0.926 |
| 4-5 mm | **6-7 mm** | -1.667 | 1.121 | 0.137 | -3.868 | 0.533 |
| 4-5 mm | **7-8 mm** | -2.259 | 1.073 | 0.036^*^ | -4.364 | -0.153 |
| 4-5 mm | **8-9 mm** | -2.322 | 1.687 | 0.169 | -5.632 | 0.988 |
| 5-6 mm | **6-7 mm** | -0.741 | 1.241 | 0.550 | -3.176 | 1.693 |
| 5-6 mm | **7-8 mm** | -1.332 | 1.197 | 0.266 | -3.682 | 1.017 |
| 5-6 mm | **8-9 mm** | -1.396 | 1.768 | 0.430 | -4.866 | 2.074 |
| 6-7 mm | **7-8 mm** | -0.591 | 1.342 | 0.660 | -3.224 | 2.041 |
| 6-7 mm | **8-9 mm** | -0.655 | 1.869 | 0.726 | -4.322 | 3.013 |
| 7-8 mm | **8-9 mm** | -0.063 | 1.841 | 0.973 | -3.675 | 3.548 |

^†^LSD post hoc correction.

^*^*P* <0.05.

SE = standard error; mean difference = mean axial length of subgroup 1 – mean axial length of subgroup 2.
